# On Dropsy after Scarlatina

**Published:** 1855-02

**Authors:** O. C. Gibbs

**Affiliations:** Perry, Lake county, Ohio


### *On Dropsy after Scarlatina.*

By O. C. Gibbs, M. D., of Perry, Lake county, Ohio.

Few diseases have more troublesome or a greater variety of sequelæ than scarlet fever.

Though the case may be a mild one, and the period of the fever and rash passed with safety, death may ensue from subsequent evils; or, if life be preserved, changes may be wrought in the delicate organism that may be seen and felt throughout the subsequent period of the patient's history.

When the rash declines, and the anxiety of friends is lessened, swelling, suppuration, abscess of the parotid and submaxillary glands may supervene, and the consequent irritation and purulent discharge may exhaust the weakened system beyond the reach of its feeble recuperative energies, and having outlived scarlet fever the patient may die of hectic. Coupled with or independent, as the case may be, of this glandular disease, may occur inflammation of the internal ear, with subsequent copious purulent discharge, resulting in hardness of hearing or deafness, which will hopelessly continue for the remainder of life.

The ear is not the only organ of sense liable to injury from these troublesome sequelæ. Chronic opthalmia may come on, continuing, perhaps, for months and years, with permanent impairment of the delicate organ of vision; or inflammation of the pituitary membrane may take place, with purulent discharge from the nostrils, resulting, perhaps, in ozœna or an unpleasant and obstinate derangement of this important organ of sense.

During the decline of scarlet fever, inflammation of the mucous membranes occasionally occurs, giving rise to bronchial, gastric and enteric diseases, which may continue for an indefinite period of time or early result in death. The serous membranes, also, are by no means exempt; pleuritis, peritonitis, and worse than all, meningitis, simple or tubercular, with their concomitant dangers to life, may result from the progress or decline of this affection.

The catalogue of sequences is by no means complete; but the object of this paper was not to enumerate them or their dangers, but to give expression to the best received opinions upon the pathology and treatment of the more common and serious of them all, I mean dropsy, in those forms which succeed to scarlatina.

Dropsy, following scarlet fever, presents itself in two distinct etiological conditions. The first, most common and dangerous, has its origin in a congested or inflamed condition of the kidney. The second, less common and more amenable to treatment, owes its existence to anæmia.

1st. *Hydrops nephriticus,* or renal dropsy, may come on at different intervals after the commencement of the disease, of which it is a sequela; but, more commonly, within two weeks after the appearance of the eruption. It is usually preceded by two or three days of interrupted convalescence, indicated by impaired appetite, increased thirst, constipated bowels, scanty urine, dry and hot skin, and accelerated pulse. These signs of constitutional disturbance are accompanied by an arrest of the process of desquamation. After two or three days continuance, œdema of the face comes on, which usually awakens but little anxiety until, sooner or later, this puffiness extends to the hands and feet. These symptoms are accompanied by scanty and albuminous urine. The albuminous character, it is said, is not always present, yet upon this point there seem to be different opinions entertained. In mild cases, after a few days continuance of the above abnormal symptoms, the function of the kidneys is restored, the anasarca gradually disappears, convalescence is re-established and progresses without further interruption. In severe cases, in addition to the symptoms given above, pain and tenderness are felt in the lumbar region; the urine is very scanty, highly albuminous and frequently bloody; the œdema extends throughout the cellular tissue of the skin and lungs; the features become distorted and the limbs greatly swollen by the anasarca. But at this stage of the difficulty, the pectoral symptoms are usually the most distressing and dangerous. Cough, difficult and hurried respiration, accompanied with copious frothy expectoration, continue to harass the patient until death takes place, in consequence of the œdema of the lungs, or until these distressing symptoms pass away with the general anasarcous condition. The greater the œdema, the less the effusion into the serous cavities, and *vice versa.* Ascites, perhaps, is an exception to this. The relative frequency of dropsy of the serous membranes, is in the order named: Ascites, Hydrothorax, Hydrocardium and Hydrocephalus. These affections may occur either simultaneously or successively. I once saw a patient affected with œdema, on the subsidence of which, ascites was developed, subsequently hydrothorax, and finally hydrocephalus, of which the patient died. When effusion takes place into any of the serous cavities, the symptoms peculiar to each of these several dropsies are superadded to those given above.

In these cases, the energy of the system seems to be overwhelmed with the intensity of the diseased action, the pulse is very much accelerated and enfeebled, respiration is labored, the urine is not only very scanty but brown, or reddish brown, from sanguineous intermixture, and unless the kidneys are speedily restored to their normal function, with a corresponding abatement of the dropsical symptoms, death may take place from an increase of the pectoral disease; from the effects of effusion into some of the serous cavities; or from coma resulting from the poisonous properties of the urea retained in the circulation.

(*a.*) Of the *nature and cause* of that form of renal dropsy which succeeds scarlet fever, there has been expressed a diversity of opinion. It is highly probable that the congestion of the kidney, and the exfoliation of the epethelial cells, are as much the specific effect of scarlatina poison, as is the eruption upon the skin and the cuticular exfoliation.

It is said dropsy is more frequent after a mild than a severe case of scarlet fever; or, rather, is more likely to succeed those cases accompanied with but an insignificant eruption. The reason of this is apparent, if we admit the specific nature of the renal affection; for the more perfect the elimination of the scarlatina poison by the skin, the less the tax upon the kidneys. This view derives support, when compared with the reciprocal relation existing between the catarrhal and eruptive symptoms in rubeola. Every one knows that the catarrhal symptoms in measles, are as much a specific effect of the rubeolous poison, as is the eruption itself; and, if the rash is tardily or but imperfectly developed, the catarrhal symptoms are fearfully increased, and bronchitis or pneumonia is usually induced.

Most writers agree in considering cold the most common and prolific cause of effusion in scarlatina. But if the specific nature of the renal affection be admitted, then the albuminous character of the urine is not produced by cold.

Effusion will probably not take place, so long as the amount of the retained urea is within the bounds of the accommodating power of the system. If this be so, then anything that will diminish the activity of the organism or increase the amount of the retained urea beyond this accommodating power, becomes the immediate cause of the dropsical symptoms.

(*b.*) The *pathology* of this affection, it is not here proposed minutely to consider. From what has been said, it is apparent that we must look to the kidneys, would we find those pathological lesions which give rise to that form of dropsical disease now under consideration. An examination after death reveals the kidneys enlarged, generally darker and more congested than natural. Their surfaces usually present a pale and mottled appearance, which contrasts, in a marked degree, with the internal tubular parts of the cortical structure. The pelvis and infundibula are involved, generally, in the increased vascularity, but by no means uniformly so. Diligent microscopical researches have shown that the cortical portions are the first to give evidence of the morbid process, and more particularly, the urinary tubules. "This congestion," observes Dr. Behrend, "induces exudation and hemorrhage from rupture of the capillaries, both effusions mingling with the urine, which becomes of a dark chocolate color. By means of the exudation, the small vessels and tubuli of the cortical substance are in part obliterated, and in part compressed, a granular appearance resulting under the microscope; the capillaries are observed in part empty, and in part filled with exudation globules, without any red blood; and hence, assuming a whitish-straw color. A yet stronger pressure of blood and urine produces, within the tubuli, a separation and regeneration of epithelium, analogous to the eruption and desquamation of the surface; and, finally, albumen is separated with the urine."

This impaired activity of the cortical substance, this morbid condition of the urinary tubules and epithelial cells, prevent the proper elimination of the urea, and the retention of this substance, in connection with the scarlatina poison, acts efficiently in the production of the troublesome sequelæ under consideration.

(*c.*) The *mode* in which dropsy is induced from the above described renal affection, is, perhaps, the following : The retained urea acts as a poison, deteriorating the blood, from which retained excretion the system attempts to relieve itself, perhaps by an increase of the other secretions. Succeeding in this, no dropsy is produced. But if the amount of the retained urea is too great, or its deficient elimination too long continued, for the successful resistance of the enfeebled powers of the system, excitement of various parts and sundry effusions ensue.

Besides this excrementitious retention, there is a deficiency of albumen in the blood, which loss, by thinning that fluid, predisposes to and facilitates dropsical effusions. It is to this impairment of the circulating fluid, from the causes mentioned, and the disposition of the scarlatinous poison to become eliminated by the skin, that dropsical effusions are probably due ; the effusions into the serous membranes being attributable to the former cause alone.

2d. *Hydrops anœmicus*, or dropsy produced as a consequence of anæmia, occasionally succeeds scarlet fever. The renal affection above described—the deficiency of albumen, and the retained urea in the blood—may so deteriorate that fluid, by diminishing the red globules and increasing the watery constituents, as to resemble anæmia. But by anæmic dropsy is meant that form of effusion, resulting from a deficiency in quantity and deterioration in the quality of the blood, independent of any renal affection or marked morbid condition of the urine. The diminished and impoverished condition of the blood in anæmia disturbs circulation, promotes congestion or local determination of blood, and facilitates watery effusion. The heart, failing to receive its normal amount of healthy stimulus, performs its action less perfectly than in health. This impaired circulation, in connection with a lax condition of the veins, predisposes to congestion and congestion to effusion. Not only so, but in debility, the relaxed condition of the capillary blood vessels allows the liquid portion of the blood to pass through their walls with less than ordinary resistance.

In the *treatment* of renal dropsy, resulting as a sequela of scarlatina, the indications of treatment are, 1st, to relieve the congested condition of the kidneys; 2nd, to restore the normal functions of these organs; and 3d, to support the system against the prostrating effects of deteriorated blood.

1st. To relieve the congested condition of the kidneys, blood-letting, purgatives and diaphoretics are the instrumentalities most to be relied upon. In the early stage, when the symptoms of congestion are well marked, when the urine is acid, blood-colored and albuminous, blood may be taken from the arm, but not with that freedom which is justifiable in most other congestive diseases. The reason of this is obvious, when it is remembered that the patients are mostly young children, and that the retained urea, in connection with the scarlatinous poison, rapidly deteriorates the blood and induces anæmia. Venesection is a remedy of such power in such cases, that without the exercise of much wisdom as to the case, to time and quantity of the abstraction, more of harm than good may result. In some cases, cupping to the small of the back or leeches applied in the region of the kidneys, may be a safer means of depletion. But in most cases, probably the loss of blood can be wholly dispensed with, trusting to the anti-phlogistic and revulsive effect of cathartics and diaphoretics. Of cathartics, the saline are to be preferred, and may be administered daily or less frequently, as the circumstances of the case may require. Of diaphoretics, antimony should be preferred in cases of great vascular excitement, accompanied with considerable head-ache, and a very hot and dry skin. The neutral mixture and the solution of the acetate of ammonia, are appropriate remedies in such cases. The Dover's powder may be given in such cases as are unaccompanied with headache or a disposition to stupor. These may be given singly, or variously combined to meet the peculiarities of the age, vigor of constitution, period of disease or intensity of morbid action. Whatever be the choice or combination of diaphoretics, the medicine should be given every two or three hours, so as to maintain an uninterrupted impression. The daily administration of the warm bath, is a powerful auxiliary to the above mentioned remedies.

2d. To restoring the normal function of the kidneys, by the administration of diuretics, high authority stands opposed; or, at least, there is an honest diversity of opinion among medical writers, in regard to the propriety of such a procedure. But it is presumable that objection is mostly made upon theoretical grounds. Many suppose that diuretics, by stimulating the kidneys, increase their already congested condition, and thus defeat the very object for which they are administered. In measles, small pox, or scarlet fever, we do not hesitate to administer diaphoretics, notwithstanding the congested condition of the skin ; neither do we fear any increase in the intensity of disease by such administrations. But, on the contrary, it is upon such medicines that we place our greatest reliance.

Admitting the specific nature of the congestion and exfoliation of the epithelium, lining the tubuli of the cortical structure of the renal organs, why should diuretics be more inappropriate than diaphoretics in congestion and exfoliation, upon the surface of the body in uncomplicated cases of scarlatina ? Again : when we consider that the greatest danger in these cases, arises from the retained urinary excretions, and that improvement is simultaneous with the return of the normal functions of the kidneys, the importance of the judicious administration of diuretics becomes an almost irresistible conviction. Of diuretics, cream of tartar and digitalis are probably superior to all others, excepting in cases of extreme debility. These may be given singly or in combination, with decided advantage, and without risk of increasing renal congestion, if the dose and frequency of repetition be proportioned to the age of the patient and the varying symptoms of individual cases. Many careful and judicious practitioners, would probably advise diuretics, only after several days' continuance of the antiphlogistic remedies, mentioned when speaking of the accomplishment of the first indication. But, probably, in most cases, after general or local bleeding, as the case may require, or after the administration of a saline cathartic, should loss of blood be considered injudicious, the diuretics above mentioned may be commenced with at first, and continued in appropriate doses every three or four hours, in connection with an occasional cathartic.

3d. When the urine is of a whitish-yellow color, from its large proportion of albumen, and ceases to exhibit an acid reaction, antiphlogistics must be dispensed with, and the rapid deterioration of the blood guarded against by the judicious administration of tonics. Diuretics may still be persevered in, and there is nothing inconsistent in giving tonics in connection with cream of tartar and digitalis. But, if the anæmia is very considerable, the more stimulating diuretics may be preferred. The spirits of nitre is particularly appropriate in cases accompanied with nervous derangement. Of the tonics, perhaps the muriated tincture of iron is equal, if not superior, in these cases, to any other known.

The treatment of anæmic dropsy consists, essentially, in the administration of tonics and stimulating diuretics. A judicious administration of the remedies mentioned, both in reference to the age of the patient and the peculiarities of each case, will, in a great majority of cases, eventuate in recovery.

